# A Review of the Compressive Stiffness of the Human Head

**DOI:** 10.1007/s10439-022-03099-5

**Published:** 2022-11-12

**Authors:** Darcy W. Thompson-Bagshaw, Ryan D. Quarrington, Claire F. Jones

**Affiliations:** 1 School of Mechanical Engineering, The University of Adelaide, Adelaide, SA Australia; 2 Centre for Orthopaedic & Trauma Research, The University of Adelaide, Adelaide, Australia; 3 Adelaide Spinal Research Group, Adelaide Medical School, The University of Adelaide, Adelaide, SA Australia

**Keywords:** Impact response, Head injury, Cephalus, Head impact, Head form

## Abstract

Synthetic surrogate head models are used in biomechanical studies to investigate skull, brain, and cervical spine injury. To ensure appropriate biofidelity of these head models, the stiffness is often tuned so that the surrogate’s response approximates the cadaveric response corridor. Impact parameters such as energy, and loading direction and region, can influence injury prediction measures, such as impact force and head acceleration. An improved understanding of how impact parameters affect the head’s structural response is required for designing better surrogate head models. This study comprises a synthesis and review of all existing *ex vivo* head stiffness data, and the primary factors that influence the force–deformation response are discussed. Eighteen studies from 1972 to 2019 were identified. Head stiffness statistically varied with age (pediatric vs. adult), loading region, and rate. The contact area of the impactor likely affects stiffness, whereas the impactor mass likely does not. The head’s response to frontal impacts was widely reported, but few studies have evaluated the response to other impact locations and directions. The findings from this review indicate that further work is required to assess the effect of head constraints, loading region, and impactor geometry, across a range of relevant scenarios.

## Introduction

Synthetic surrogate head models, such as anthropomorphic test devices (ATD; e.g. Hybrid III, FOCUS and NOCSAE) or custom head models,^11,37,38^ are used in experimental models of head and head-neck injury events to assess the risk of skull, brain and/or cervical spine injury.^30,38,43^ To enable accurate prediction of injury, these head models should possess an impact response that lies within cadaveric response corridors (mean ± standard deviation) for the relevant test configuration.^16,33^ The most commonly used surrogate head model, the Hybrid III, was designed to replicate the human head’s response to frontal impacts by benchmarking it against acceleration data from a series of embalmed, cadaveric head impacts.^13,26^ Despite its widespread use in biomechanical research, the Hybrid III impact response does not compare well to the cadaveric response in facial,^1^ vertex, or parietal^22^ impacts. Accurate characterization of the response of the human head to all injury-relevant loading scenarios is required to design improved surrogate head models.

The structural response of the human head to an external force is usually described by a force–deformation relationship. This relationship comprises an initial toe-region corresponding to skin deformation, and a linear region corresponding to skull deformation.^40^ In studies of the isolated skull (without soft tissue), slope of the linear region was dependent on the rate and region of the applied load,^18,29^ likely due to viscoelastic response of bone tissue, and variation in bone thickness, curvature and density, respectively.

To design surrogate heads with an adequate biofidelic mechanical response, an understanding of the factors that influence the force–deformation relationship for the whole head is needed. *Ex vivo* studies of the mechanical response of the head have been partially summarized in reviews concerning skull fracture tolerance^3,45^ and bone motion during cranial osteopathy^40^; however, a review of human head stiffness data is not available. The aim of this study is to synthesize the existing *ex vivo* human head stiffness data, and to explore the influence of the loading rate and region and the experimental boundary conditions on this stiffness data.

## Methods and Materials

Publications prior to July 2022 that reported the force–deformation relationship of the human head were identified by searching PubMed with the following search terms: skull AND (quasi OR dynamic) loading AND (deformation OR deflection). Further articles were identified *via* the citation lists of these primary publications. Studies in which the load was applied to the mandible or neck, or in which the force–deformation data were not reported, were excluded.

The mechanical response of human heads was reported in eighteen studies (14 manuscripts, 2 published conference proceedings, and 2 theses), published between 1973 and 2019. Two studies were excluded as the head was impacted by a small, high velocity, ballistic^36^ or the head impacted a padded surface^47^ and the isolated head response was not reported. Twelve studies evaluated the response of intact fresh-frozen heads, and four studies^23,28,41,42^ used either dry skulls and/or embalmed heads. Papers with the latter two specimen categories were retained only for consideration of their experimental methods, as embalming and drying processes significantly affect the mechanical response of bone.^8,24,32^ The studies using fresh-frozen tissue were categorized according to the use of local (9 studies) or global compression loading (3 studies). Local compression loading was defined as using an external object to load a single region of the head. Global compression loading was defined as uniaxial compression applied *via* a large, parallel surfaces that simultaneously compressed the anterior and posterior surface, or bilateral surfaces, of the head, representing an uncommon “crushing” trauma. Local compression loading studies were further categorized into frontal loading only (4 studies), or varied loading region (5 studies).

## Results

### Overview of Experimental Methods

Across quasi-static to dynamic loading rates, the force–deformation relationship was evaluated with heads loaded by materials testing machines (4 studies), drop towers (2 studies), pendulums (1 study), free-falling techniques (2 studies), and unconstrained projectile apparatus’ (2 studies). In all studies uniaxial force was measured with a load cell attached to the loading surface, or fixed between the specimen and its supporting structure. In most studies, uniaxial deformation was measured using a displacement sensor (linear variable differential transformer, string potentiometer, or laser distance sensor) fixed to the loading apparatus. For free-falling and unconstrained projectile methods, deformation was determined by double integration of an acceleration-time signal, which was recorded with an accelerometer fixed to the head or impactor,^4,6,37,41^ or calculated by normalizing the force–time signal with the head mass.^20,22^

### Global Compression

**Figure 1:**
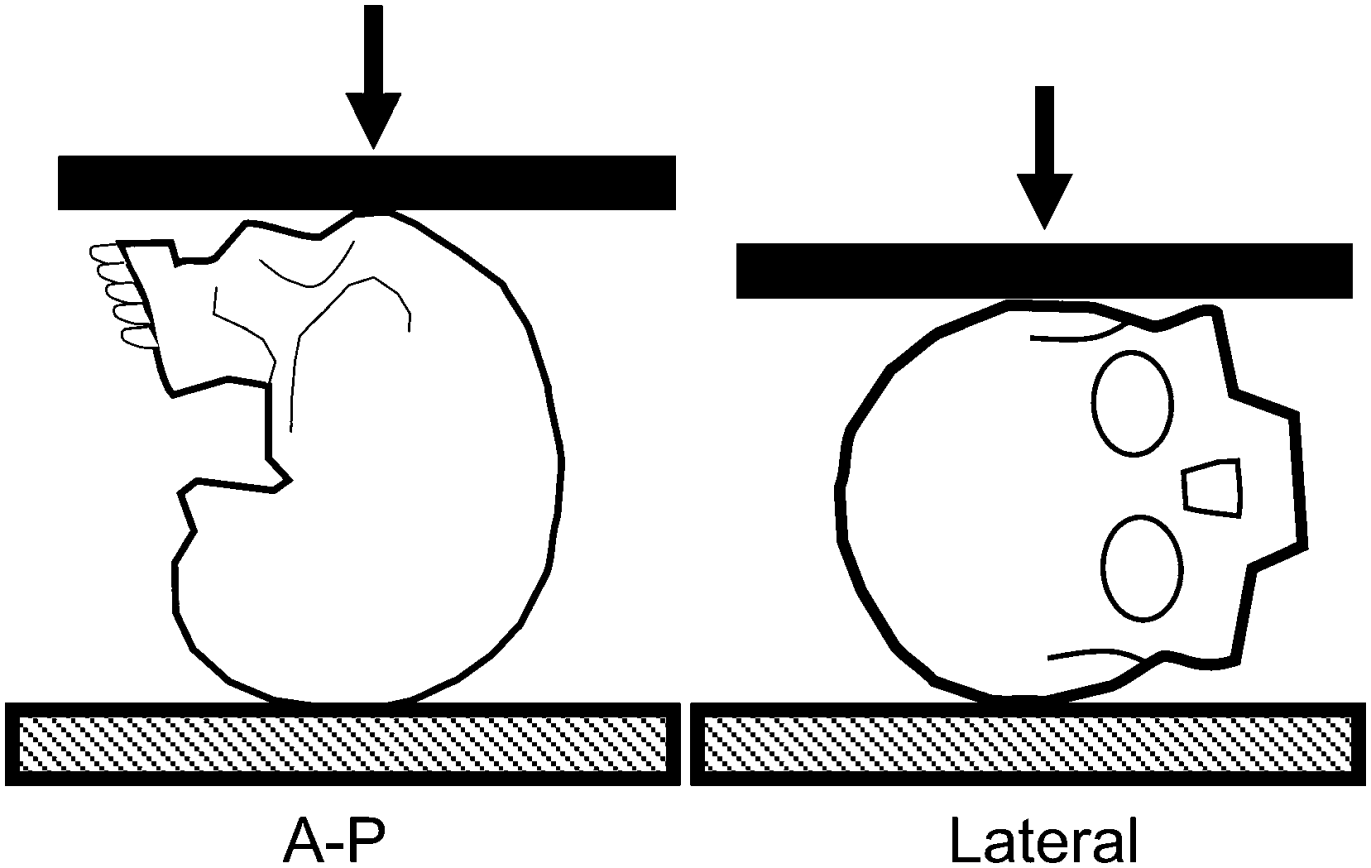
Test setup schematics for heads compressed in the anterior–posterior (left) and lateral (right) loading directions.

The force–deformation response of the adult^25^ and pediatric^35^ head were first evaluated in 1972 and 2004, respectively. These studies applied low-rate compression loads in the anterior–posterior (A-P) and lateral direction (Fig. 1) to identify loading rate or direction dependencies of the head’s structural response.

McElhaney *et al*.^25^ placed adult heads between parallel platens and applied destructive quasistatic compression loading *via* a material testing machine. Relative to the lateral loading direction, A-P loading (*N* = 12/direction) demonstrated similar peak force (5954 ± 1869 vs. 5155 ± 1183 N; *p* > 0.05, see Footnote 1; Table 1), lower deformation (3.8 ± 0.9 vs. 6 ± 1.6 mm; *p* < 0.001^1^), and higher stiffness (2450 ± 1052 vs. 1222 ± 526 N; p < 0.001, see Footnote 1). These findings indicate a directional dependence of the head’s response to destructive quasistatic compression.

**Table 1:** Summary of the methods and stiffness results for studies that applied anterior–posterior (A-P) and lateral compression to human heads.

Methods	Donor info		Loading limits	Loading rate (mm/s)	Mean stiffness (N/mm)	
	*N*	Age			A-P	Lateral
Destructive compression of adult heads^25^	*N* = 12 per group (21 males)	69 ± 13 years	–	–	2450 ± 1052	1222 ± 526
			Load limit: 500 N	0.05–0.01	26 ± 39	23 ± 21
	*N* = 12 (5 males)	20 weeks–16 years	Displacement limit: 5% of head width (lateral, mean 5.4 mm) or length (A-P, mean 7 mm)	0.7–1.9	43 ± 56	33 ± 23
				7–19	53 ± 69	43 ± 27
Repeated, non-destructive compression of pediatric heads^a^^21,35^				22–57	70 ± 93	47 ± 30
			Load limit: 1000 N	0.08–0.1	308^b^	385^b^
Repeated, non-destructive compression of adult heads^19^	6 males	60 ± 5 yrs	Displacement limit: 5% of head width (lateral, mean 7.6 mm) or length (A-P, mean 9.8 mm)	1.5–2.1	737^b^	409^b^
				15–21	1036^b^	583^b^
				46–63	853^b^	644^b^

^a^Mean and standard deviation excludes 9 and 16 year old (*N* = 1 per age), due to the lack of data across test conditions

^b^Small-compression stiffness values, calculated between 1.25 and 2.5% of the gauge length. Means without standard deviations were obtained from reported bar graphs^19^

A series of A-P and lateral non-destructive compression loads were applied to adult (*N* = 6)^19^ and pediatric (*N* = 12)^21,35^ heads. Loading rates were normalized by the head length and width, respectively for A-P and lateral tests, to produce consistent strain rates (0.0005, 0.01, 0.1, 0.3 1/s). Load and displacement limits were set for the adult (1000 N, 5% head length/width) and pediatric (500 N, 5% head length/width) heads to prevent skull fracture. Small-compression (6.25–50% peak deformation) and large-compression (50–100% peak deformation) stiffness was evaluated for pediatric heads, but only small-compression stiffness was determined for the adult heads (Table 1). Across the five age groups (premature, neonate, toddler, youth, and adult), generalized linear models showed that the small- and large-compression (excluding adults) stiffness was dependent on age, but independent of loading rate and direction.^19^ The adult stiffness findings contradict the previous destructive study,^25^ but it is likely that the low severity, non-destructive response was primarily influenced by the response of soft tissue and not bone.

### Varied Local Region Compression

Four studies compared the head’s response to loading applied across a number of regions. Two studies^2,46^ correlated biomechanical parameters (force, deformation, stiffness) with the presence of fractures to further understand the regional differences in the head’s response, as these differences may have implications with injury prediction measures (e.g. head injury criteria) and in clinical applications.^45^ The remaining two studies^20,22^ assessed the regional differences for non-destructive impact properties of adult and pediatric heads, and compared the impact response (stiffness, pulse duration, acceleration and HIC) against the response for age-matched surrogate heads. Overall, the head’s response was shown to be region- and rate-dependent.

In one study, heads were fixed to the base of a drop tower and impacted by a circular (lateral region; 2.7 m/s; *N* = 11) or rectangular (parietal region; 4.3 m/s; *N* = 20) plate.^2^ Specimens were partially embedded in plaster in the right lateral recumbent position (lateral impacts) or rotated 45° to the horizontal plane (parietal impacts; Table 2). Stiffness was greater for the parietal impacts (4168 ± 1626 N/mm) compared to the lateral impacts (1799 ± 881 N/mm). The authors noted that stiffness appeared to be affected by the contact area, but the response was likely influenced by the different loading-rate, impactor contact area and region, and head constraints.

**Table 2:** Summary of the methods and force, deformation and stiffness results for studies that compared the impact the head at various loading region.

Test setup	Donor info	LR (m/s)	IE (J)	Peak force (N) ∣ Deformation at peak force (mm) ∣ Stiffness (N/mm)				
				Vertex	Parietal	Frontal	Lateral	Occiput
Allsop *et al*.^2^	*N* = 20	2.7	39	–	–	–	5400 ± 1984^a^	–
	8 males			–	–	–	–	–
	70 ± 13 years			–	–	–	1799 ± 881	–
	*N* = 11	4.3	111	–	12390 ± 654^a^	–	–	–
	4 males			–	–	–	–	–
	58 ± 20 years			–	4168 ± 1626	–	–	–
Yoganandan *et al*.^46^	*N* = 12^c^ 4 males 67 ± 8 years	0.0025	–	4464^a^	5604^a^	4645^a^	6182^a^	11989^a^
				9.1	8.4	14.1	15.4	16.6
				790	919	467	487	1290
		7.1 – 8	–	12004^a^	–	13600^a^	–	10009^a^
				3.5	–	5.9	–	4.0
				3953	–	5867	–	2462
Loyd *et al*.^22^	6 males 60 ± 5 years	1.71	4.8	4587 ± 368	3319 ± 551^b^	3656 ± 605	–	3785 ± 309
				–	–	–	–	–
				3065 ± 483	1973 ± 589^b^	2084 ± 541	–	2182 ± 441
		2.42	9.6	6828 ± 797	4945 ± 870^b^	5771 ± 930	–	5775 ± 835
				–		–	–	–
				3699 ± 754	2162 ± 715^b^	2918 ± 905	–	2959 ± 935
Loyd *et al*.^20^ Same schematic as above	*N* = 12 5 males 33 week gestation–16 years	1.71	2	816 ± 858	709 ± 878^b^	762 ± 882	–	841 ± 1060
				–	–	–	–	–
				390 ± 665	273 ± 480^b^	273 ± 428	–	343 ± 631
		2.42	4	1219 ± 1448	1006 ± 1167^b^	1065 ± 1162	–	1349 ± 1779
				–	–	–	–	–
				392 ± 752	306 ± 470^b^	266 ± 370	–	498 ± 926

Data are given as mean ± SD where available.

*LR* loading rate, *IE* input energy

^a^Fracture force

^b^Stiffness results from left and right parietal impacts were combined

^c^*N* = 1 for quasistatic vertex, frontal, lateral and occiput, and dynamic frontal and occiput. *N* = 2 for quasistatic parietal and *N* = 4 for dynamic vertex

Using a hemispherical anvil, quasistatic (2.5 mm/s; *N* = 6) and dynamic (7.1–8 m/s; *N* = 6) compression loads were applied to five regions (vertex, frontal, right parietal, right temporal, or occiput) of heads that were rigidly supported at the base, until failure occurred.^46^ The force–deformation relationship exhibited a local maxima prior to the peak load for quasistatic, but not dynamic, loading rates. Comparisons between loading regions are limited by the low number of specimens per region for a given rate (usually *N* = 1); however, across these regions stiffness was lower for quasistatic (467–1290 N/mm) than dynamic (2462–5867 N/mm) loading rates (Table 2).

Three papers reported the mechanical response of adult^22^ and pediatric^20,35^ heads to repeated 15 and 30 cm free-falling impacts (1.71 and 2.42 m/s), at five regions (in order: forehead, occiput, vertex, right parietal, left parietal). In both studies, the following outcomes were supported by statistical significance: adult head stiffness was lower for parietal impacts than vertex impacts; adult and pediatric head stiffness increased with loading rate (Table 2); and pediatric head stiffness increased with age.

### Local Frontal Region Compression

Four studies assessed the impact response of the frontal and facial regions to compare and validate the Hybrid III^1,22^ or FOCUS^4,6^ surrogate heads, and one study^9^ investigated a potential energy-to-failure criterion for frontal bone impacts.

Allsop *et al*.^1^ reported the response of heads (*N* = 13) impacted sequentially at two locations: the midface (maxilla or zygoma) and the frontal bone; with the assumption that initial fractures did not influence the subsequent response. Heads were fixed in plaster in the supine position, with the Frankfort plane vertical and elements posterior to the frontal plane embedded (Table 3). The heads were impacted (3–4.2 m/s) by a semicircular rod, which spanned the width of the head and was attached to a 14.5 kg carriage. Compared to the facial impacts, the reported mean frontal bone fracture force and stiffness were greater (Table 3).

**Table 3:** Summary of the test setup and fracture force, deformation at fracture (D), and stiffness results (mean ± std) for studies that loaded the frontal region of adult human heads.

Authors	Test setup	Donor info		Region	LR (m/s)	IE (J)	Fracture force (N)	D (mm)	Stiffness (N/mm)
		*N*	Age (years)						
Allsop *et al*.^1^		13, 4 males	73 ± 13	Maxilla	3–4.2	65–128	301 ± 142	–	142 ± 59
				Zygoma	3–4.2	65–128	1737 ± 148	–	472 ± 52
				Frontal	3–4.2	65–128	4715 ± 1667	–	1013 ± 500
Cormier *et al*.^6^		25, all males	72 ± 18	Frontal	5.3	47	1982 ± 765	–	978 ± 523
				Nasal	2.2	8	670 ± 201	–	261 ± 217
				Maxilla	2–5	8–44	1057 ± 551	–	360 ± 142
Brozoski^4^		25, all males	Not reported	Frontal	4–6	26–64	1994 ± 909	–	683 ± 191
				Zygoma	4	29	905 ± 552	7^a^	208 ± 73
				Nasal	2.1–2.5	7–9	143 ± 103	4 ± 1.2	40 ± 12
Deyle *et al*.^9^		1 male	73	Frontal	3.6	214	5938	3.5	2931^b^
		8, 4 males	80 ± 6	Frontal	5.2	590	11069 ± 2512	5.1 ± 1.1	3165^b^
		4, 2 males	79 ± 10	Frontal	6.9	696	10238 ± 2218	4..3 ± 0.2	4769^b^

*LR* loading rate, *IE* input energy

^a^Standard deviation not reported

^b^Stiffness was calculated using data extracted from the reported force-deformation curve

Cormier *et al*.^6^ impacted the maxilla, nasal and frontal bone (5.3 m/s, *N* = 27) in the A-P direction while heads were constrained using similar embedding methods to the previous study^1^ (Table 3). Loading was applied *via* a small circular platen (3.2 kg, 28 mm diameter) to ensure that only a single anatomical region was loaded. Acoustic emission sensors were used to identify the time of fracture^5^; the results showed that head was capable of load bearing beyond fracture, as fractured initiation occurred approximately half of the peak force. The maxilla and nasal stiffness were similar (261 ± 217 vs. 360 ± 142 N/mm), but the maxilla fractured at a greater load (1057 ± 551 vs. 607 ± 201 N). The frontal bone was stiffest (978 ± 523 N/mm), and fractured at the highest load (1993 ± 909 N); however, the mean data were lower than that of the previous study,^1^ potentially due to the different impactor geometry and lower impactor mass.

Brozoski^4^ evaluated the response of the nasal, zygoma, and frontal bone to right-lateral impacts, in a study subsequent to that of Cormier *et al*.^6^ The frontal bone (*N* = 20) was impacted prior to either the nasal then zygoma (*N* = 10), or zygoma then nasal (*N* = 10) impacts. Similar to the response in the A-P direction,^6^ the frontal bone was stiffer and fractured at greater forces than the facial regions (Table 3). Frontal region stiffness and fracture force were similar to the A-P loading direction,^6^ but the nasal region was less stiff and fractured at a lower force (Table 3).

Deyle *et al*.^9^ impacted the frontal bone of heads with a large circular plate, using a custom, dual-arm pendulum. The heads were rigidly fixed to the apparatus *via* a steel post that was constrained at the foramen magnum. An initial impact at one of three velocities (3.6 m/s, *N* = 1; 5.21 m/s, *N* = 7; or 6.95 m/s, *N* = 4) was performed, and if no fracture was observed then the tests were repeated at a higher velocity. Mean fracture and deformation at fracture increased from 3.6 to 5.2 m/s but decreased from 5.2 to 6.95 m/s (Table 3). Stiffness was calculated from the exemplar force–deformation graph reported for each impact velocity. Deyle *et al*.^9^ described a common trend between tests. At 3.6 m/s, a higher stiffness region was followed by a lower stiffness region (region 1: 4421 N/mm, region 2: 1440 N/mm). This bi-linear response was less prominent at 5.2 m/s (region 1: 4780 N/mm, region 2: 1540 N/mm), and at 6.95 m/s a single linear relationship (4769 N/mm) was observed, suggesting that the bilinear response was dependent on loading rate.^9^

## Discussion

**Figure 2:**
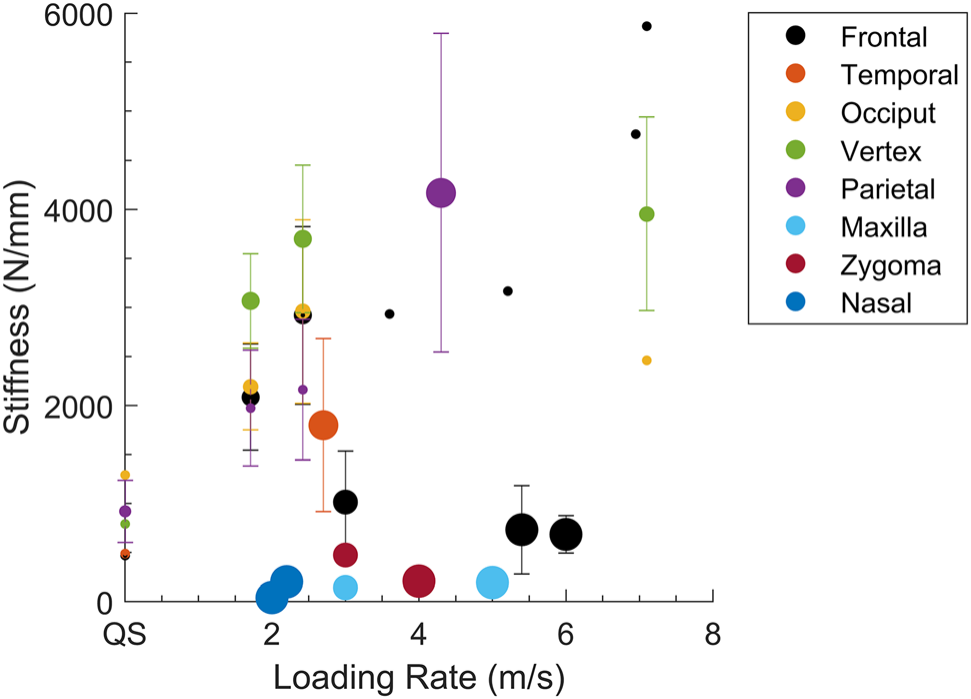
Study-specific compressive stiffness (N/mm) for fresh-frozen, adult heads in each loading region vs. loading rate or impact velocity (m/s).^1,2,4,6,9,22,25,46^ *QS* quasistatic. Markers are mean values; error bars represent one standard deviation; marker size corresponds to the number of specimens for which stiffness data is reported in the study.

Evaluating the structural response of the human head to external loads is important for the design of appropriate biofidelic surrogate head models. The stiffness of surrogate head models is often tuned to match human response corridors obtained from cadaveric studies. In a head collision event, the impactor’s energy (velocity and mass), compliance, and anatomical region of contact are known to influence the impact force and acceleration of the head^10,12,14,31^; these parameters are used to predict skull and brain injury. A better understanding of the structural response of the head will likely lead to improved biofidelity of surrogate head models. Across the reviewed studies, the stiffness of the adult human head ranged from 40 to 5867 N/mm (Fig. 2). The large variance in this response was primarily due to the region and rate of loading, but features of the loading object (mass, velocity, geometry) and experimental end-conditions likely also contributed.

### Impact Energy

As surrogate heads are used in a broad range of injury biomechanics applications, the applied impact energy can vary drastically (e.g. 18–219 J^44^). The Hybrid III head model is typically only validated against the response to a 13 J free fall impacts (375 mm, 3.5 kg head mass),^26^ but as the surrogate’s impact response is significantly affected by the impactor mass and velocity,^15^ it is unknown if the surrogate’s response demonstrates suitable biofidelity at higher energies. Understanding the effect of impact energy on the cadaveric response is needed to ensure surrogate models exhibit a suitable biofidelic response for accurate injury prediction over a range of impact energies.

Across all studies reviewed, stiffness was observed to increase with loading rate, but a consistent trend was not observed across all studies (Fig. 2). Only one study assessed the effect of impact velocity (1.7 and 2.4 m/s, non-destructive impact) on the impact response properties (stiffness, acceleration, pulse duration, and HIC),^22^ indicating it was a significant predictor for all properties. Comparing the head’s response between studies over a range of velocities, was prohibited by the large differences in testing apparatus, as these were thought to substantially alter the response. Further studies are required to investigate the effect of impact velocity over range of non-destructive and destructive velocities.

Impact mass likely does not influence the human head stiffness (assuming there is sufficient energy to exceed the toe-region), but the effect of mass on the absorbed energy (integral of force–deformation relationship) may have implications for predicting skull fractures using an energy-to-failure criterion.^27^ No study assessed the effect of impact mass on the head’s impact response. Comparisons can be made between two frontal impact studies^9,22^ that applied substantially different impact energies (2–3 m/s: 10 vs. 214 J) due to the mass of the impactors used (3.3 vs. 37 kg). Despite a 20-fold increase in impact energy, stiffness (2918 vs. 2931 N/mm) and peak force (5771 vs. 5938 N) were comparable, however, the heads only fractured in the higher impact energy group. Further studies should investigate the effects of impactor mass on the cadaveric stiffness and absorbed energy to provide surrogate head validation data over a range of energies, as well as to improve understanding of the energy-to-failure criterion.

### Loading Region

Head stiffness varied by loading region,^1,2,20–22,25,46^ but differences between regions were not consistent across impact velocities. For non-destructive impacts (1.7 and 2.4 m/s^22^), impacts to the vertex region was the stiffest compared to the occiput, frontal, and parietal regions. At intermediate impact velocities (2.7–4.2 m/s), the parietal region was the stiffest,^2^ compared to the lateral^2^ and frontal region.^1^ There are insufficient studies investigating the head’s response for the parietal, vertex, and lateral regions, particularly for destructive loading, to accurately characterize the region-specific variation of the force–deformation response.

### Experimental End-Conditions

For *ex vivo* head impact studies, the boundary and loading constraints should be applicable to the research question and representative of real-world scenarios. The geometry and rigidity of the loading surface or impactor should mimic the real-life head contact object (e.g. flat wall or steering wheel) and the head constraints should represent the physiological end-conditions to achieve realistic stress distribution throughout the tissue. With the appropriate testing apparatus, the simulated injury events should produce biofidelic loading responses, resulting in clinically relevant injuries.

#### Geometry of the Contacting Object

**Figure 3:**
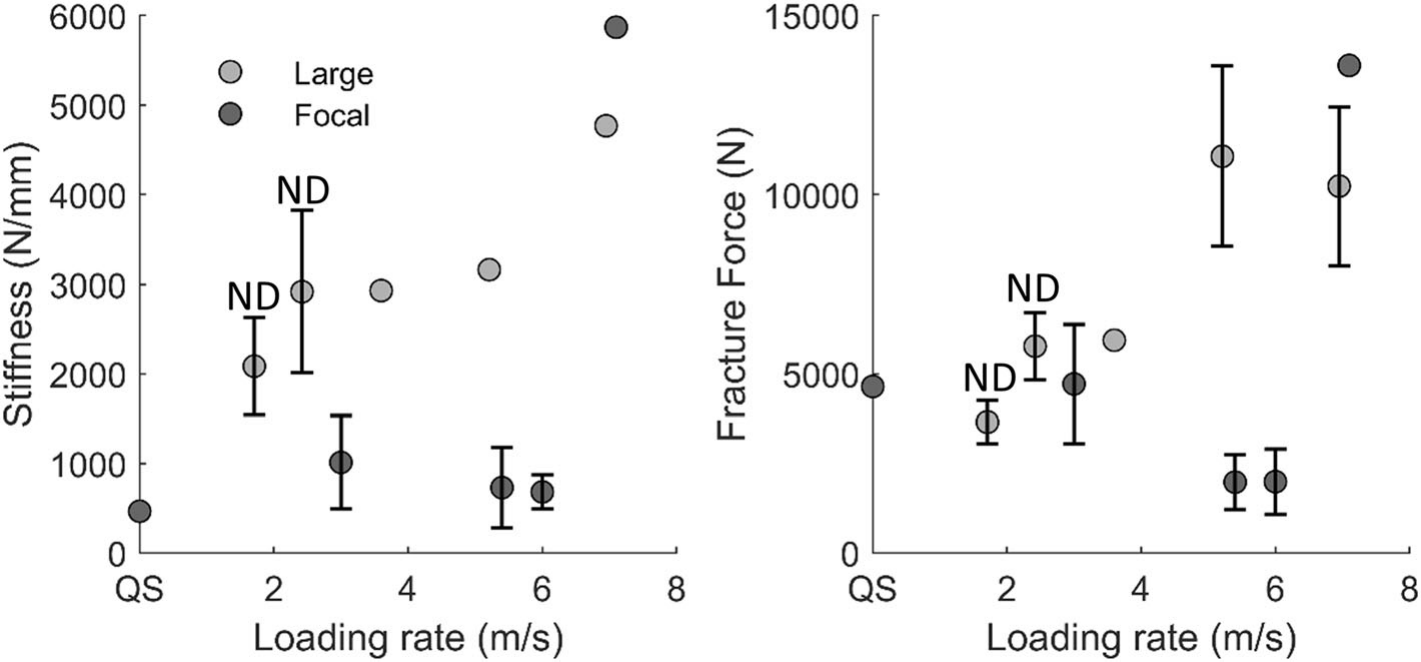
Frontal region mean (left) stiffness and (right) fracture force, vs. loading rate, with studies classified as focal^1,4,7,46^ or large^9,22^ impactor area. Error bars represent one standard deviation. *QS* quasistatic, *ND* non-destructive test.

The interaction of the contacting object and head has been shown to influence the loading response in cadaveric (without force–deformation data) and computational studies.^13,39,48^ In the reviewed studies, specimens were compressed by large or focal flat surfaces, or curved objects, but no single study explored differences in the response while varying contact geometry (area and curvature) and holding all other conditions constant. Generally for frontal region studies, lower stiffness and peak/fracture force were reported in studies with a focal contact area^1,4,6,46^ compared to a larger contact area^9,22^ (Fig. 3). Further studies are required to understand the effect of the contacting object’s geometry in other regions of the head.

#### Head Constraints

**Figure 4:**
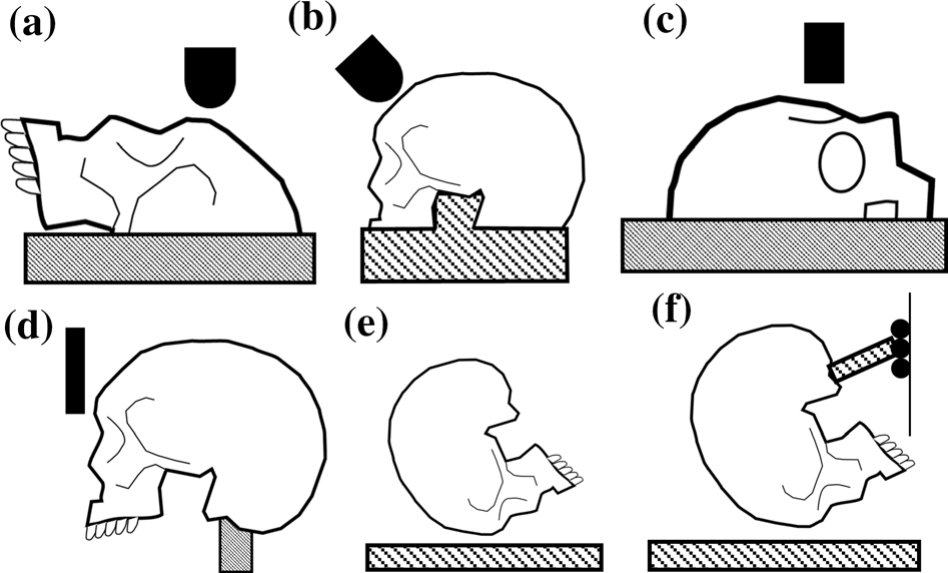
Head constraint techniques used in frontal region loading studies. The heads were partially embedded with the head (a) supine,^1,6,7^ (b) upright,^46^ or (c) right lateral recumbent.^4^ Alternatively, the heads were embedded proximal to the foramen magnum (d, f)^9,42^ or unconstrained (e).^20,22,45^

For impact studies, the heads were rigidly constrained using an embedding compound, to either partially submerge the head (*N* = 4 studies; Figs. 4a−4c),^1,4,6,7^ or to conform a steel post to the foramen magnum (*N* = 3 studies; Fig. 4d).^9,41,42^ When large portions of the heads were rigidly secured, the authors assumed that deformation would only occur at the site of loading^1,2,6,28^; however, there is little consensus on whether substantial stresses occur only at the site of loading, or propagate substantially from the location of load application.^17^ The latter head constraint technique was thought to provide a more realistic boundary condition by rigidly securing only the articular surfaces of the occiput (Fig. 4d).^41^ No study explored differences in mechanical response when altering head constraint configuration. However, representing the physiological constraints likely allows the head to deform in a more realistic manner, which should produce a more biofidelic loading response and fracture type.

In cadaveric free-fall studies the heads were unconstrained (Fig. 4e) resulting in translations and rotations prior to impact^20,22^; this likely produced an erroneous acceleration-time response (obtained *via* force data normalized by the head mass), from which deformation was calculated. Freefall experimental methods could be improved by constraining the head to a rigid surrogate neck and allowing only one degree of freedom motion, as in porcine^34^ and surrogate head freefall studies (Fig. 4f).^44^ With the heads constrained to the apparatus, local deformation can be directly measured rather than inferred from accelerometer data.

### Bilinear Loading Response

All studies reported linear and/or bilinear force–deformation relationships. For a bilinear response, the force–deformation curve progressed through an initial toe region, into a higher stiffness region, followed by a lower stiffness region until failure. This response was observed for some destructive quasistatic and dynamic tests, and with dry, embalmed and fresh-frozen heads,^42^ and was thought to arise from rate-dependent fracture mechanics of the skull.^9^ The bilinear response was absent in non-destructive^21,22,35^ and destructive^4,6^ studies that calculated deformation from acceleration-time data, which is likely a limitation of the instrumentation. For studies that measured deformation directly during frontal impacts^1,2,9,46^ the peak force was achieved in approximately one millisecond. Deyle *et al*.^9^ observed the bilinear response when sampling at 65 kHz, but the earlier studies likely captured at an insufficient rate over the short duration (2.7–4.3 m/s, 5 kHz; 5 data points^1,2^; 7.1–8 m/s, 8 kHz; 8 data points^46^). Further investigation is needed to understand the bilinear force–deflection response, as it may have implications for modelling the mechanical response of the skull during destructive loading.

### Comparison of Adult Cadaveric and Surrogate Head Model Stiffness

Four studies evaluated the stiffness of adult cadaveric and ATD heads in the same apparatus.^1,4,6,22^ One study compared the stiffness of a custom surrogate head model and cadaveric heads,^37^ but this study was not included in this review as the heads were impacted by a small, high velocity, ballistic.

**Figure 5:**
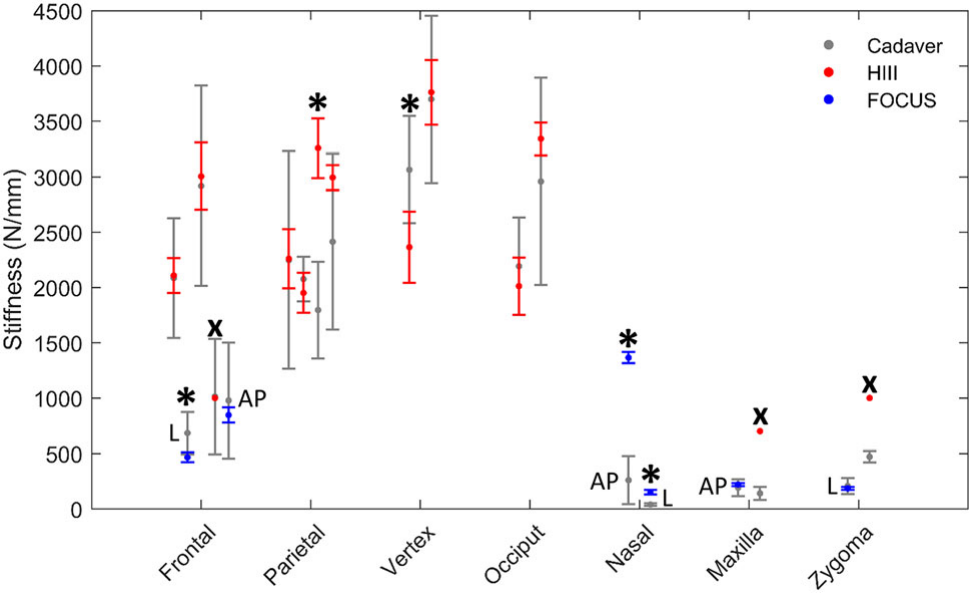
Summary of regional stiffness (mean ± SD) results for studies^1,4,6,22^ that impacted cadaveric heads and either the Hybrid III (HIII) or FOCUS head models. *Significant difference between cadaveric and ATD stiffness. ^x^Excluded from statistical comparison as only the mean stiffness for the HIII was reported. The FOCUS stiffness results have been labelled anterior–posterior (AP) or lateral (L) to distinguish impact direction.

The Hybrid III head was designed to produce a biofidelic frontal impact response, but it is often used to investigate injury risk from direct impacts to various regions of the head. Allsop *et al*.^1^ reported the frontal and midface stiffness for the adult cadaveric head and the Hybrid III head, citing similar stiffness (without statistical comparison) for frontal, but not maxilla or zygoma impacts (Fig. 5). Loyd *et al*.^22^ compared the stiffness of the adult male head and Hybrid III head, using generalized linear models with head type, impact location (frontal, occiput, frontal, left parietal and right parietal) and drop height (15 and 30 cm) as independent variables. Although stiffness was not dependent on head type (*p* = 0.12), the Hybrid III and adult male head stiffness differed for 15 cm vertex (*p* = 0.03^2^) and 30 cm right parietal (*p* < 0.001, see Footnote 2) impacts (Fig. 5). The application of these findings to the broader population in an injurious loading scenario may be limited by the non-destructive loads, small number of specimens and unisex cadaveric population. Generally, the Hybrid III compared well to destructive and non-destructive frontal impacts, and non-destructive occipital impacts, in cadaver heads. However, its response was non-biofidelic in facial, vertex and parietal impacts, and the response to lateral impacts was not reported.

The FOCUS head was designed to overcome the poor facial biofidelity of the Hybrid III head; its response was compared to adult male cadaveric data in two studies. Brozoski^4^ performed non-destructive lateral impacts (2 m/s) to the nasal bone (FOCUS *n* = 2, cadaver *n* = 19), frontal bone (*n* = 10, *n* = 24) and zygoma (*n* = 10, *n* = 17). Cormier *et al*.^6^ performed non-destructive and destructive A-P impacts (FOCUS: 2–2.8 m/s, cadaver: 1.7–5.6 m/s) to the frontal bone (*n* = 6, *n* = 20), nasal bone (*n* = 4, *n* = 19), and maxilla (*n* = 9, *n* = 29). Two-sample *t* tests (*α* = 0.05) were performed on those data in the current study to compare the stiffness of the FOCUS and adult male heads at each region for A-P and lateral impacts. Compared to frontal region cadaveric data, the FOCUS had similar stiffness in the A-P loading direction (*p* = 0.549), but it was less stiff in lateral, frontal region impacts (*p* = 0.001; Fig. 5). The FOCUS compared well to cadaveric data for midface impacts at the maxilla (*p* = 0.260) and zygoma (*p* = 0.376), but was significantly stiffer for nasal impacts in the A-P (*p* < 0.001) and lateral (*p* < 0.001) impact direction (Fig. 5). The force–deformation data presented for the FOCUS indicated increased stiffness with increasing deformation, suggesting the FOCUS will produce higher forces, compared to the cadaveric head, at greater impact severities.

The biofidelity of surrogate head models’ mechanical response to various impact loading configurations has not been comprehensively reported. The force–deformation response of the Hybrid III has not been reported for a broad range of impact conditions and locations that would induce fractures in human heads. Similar to the human head data synthesized herein, the Hybrid III response is likely influenced by the experimental end-conditions. For example, Hybrid III head stiffness was substantially lower for a high energy impact performed with the head constrained at the base of a drop tower^1^ (1000 N/mm), compared to a freefall impact^22^ (2918 ± 905 N/mm; Fig. 5). Future studies that aim to validate the response of surrogate head models should consider the impactor energy and experimental end-conditions imposed on the cadaveric and surrogate heads to produce a loading response that is representative of a real-world head impact.

## Summary

Surrogate head models are used in a broad range of experimental studies to investigate the potential for skull, brain and cervical spine trauma (e.g. References 30, 38, 43). The impact response of commonly used surrogate heads is only calibrated for specific loading conditions (e.g. Hybrid III—frontal impacts, FOCUS—facial impacts, NOCSAE—helmeted impacts), and when used in alternative scenarios the response can be non-biofidelic.^1,22^ Stiffness is a structural property derived from the force–deformation relationship that is used in the design process for surrogate heads. A review of all discoverable *ex vivo* compressive head stiffness data was performed. Head stiffness varied over an order of magnitude across the studies and was broadly dependent on region and rate of loading. The applied head constraints, and the geometry and mass of the contacting body, varied substantially between the experiments, which limited inter-study comparisons. The findings from this review indicate that further work is required to assess the effect of head constraints, loading region, and impactor geometry, on head impact mechanical response, across a range of real-world relevant scenarios. Such cadaveric response data will inform the design and validation of surrogate head models with more biofidelic force–deformation responses to a variety of direct head impacts.

## Footnotes

1 Peak force, deformation, and stiffness values were extracted from the reported force–deformation curves. Student *t* test (*p* < 0.05) were performed by author DTB to assess if the means were statistically different.

2 Statistical difference without *p* values was reported in the original publication.^19^ Two-sample *t* tests have been performed in the current study to assess if the Hybrid III and adult cadaveric head stiffness differ for these impact configurations.
